# *Clostridium difficile* infection after ileostomy closure and anastomotic failure in rectal cancer surgery patients

**DOI:** 10.1093/bjsopen/zrac026

**Published:** 2022-04-21

**Authors:** Young Il Kim, Chang Sik Yu, Yang Soo Kim, Chan Wook Kim, Jong Lyul Lee, Yong Sik Yoon, In Ja Park, Seok-Byung Lim, Jin Cheon Kim

**Affiliations:** Division of Colon and Rectal Surgery, Department of Surgery, University of Ulsan College of Medicine and Asan Medical Center, Seoul, Republic of Korea; Division of Colon and Rectal Surgery, Department of Surgery, University of Ulsan College of Medicine and Asan Medical Center, Seoul, Republic of Korea; Department of Infectious Diseases, University of Ulsan College of Medicine and Asan Medical Center, Seoul, Republic of Korea; Division of Colon and Rectal Surgery, Department of Surgery, University of Ulsan College of Medicine and Asan Medical Center, Seoul, Republic of Korea; Division of Colon and Rectal Surgery, Department of Surgery, University of Ulsan College of Medicine and Asan Medical Center, Seoul, Republic of Korea; Division of Colon and Rectal Surgery, Department of Surgery, University of Ulsan College of Medicine and Asan Medical Center, Seoul, Republic of Korea; Division of Colon and Rectal Surgery, Department of Surgery, University of Ulsan College of Medicine and Asan Medical Center, Seoul, Republic of Korea; Division of Colon and Rectal Surgery, Department of Surgery, University of Ulsan College of Medicine and Asan Medical Center, Seoul, Republic of Korea; Division of Colon and Rectal Surgery, Department of Surgery, University of Ulsan College of Medicine and Asan Medical Center, Seoul, Republic of Korea

## Abstract

**Background:**

Diverting ileostomy during resection of rectal cancer is frequently performed in patients at risk of anastomotic failure. *Clostridium difficile* infection (CDI) is reported to be frequent in patients who receive ileostomy closure with a questionable association to postoperative anastomosis leak. The primary aim of this study was to determine the incidence of CDI following ileostomy closure in patients who underwent rectal cancer surgery; the secondary aim was to assess the rate of postileostomy closure CDI in patients who presented with leakage at the original colorectal anastomosis site.

**Methods:**

Medical records of patients with rectal cancer who underwent ileostomy closure between January 2015 and December 2019 were retrospectively reviewed. All patients had previously received resection and anastomosis for primary rectal cancer with diverting ileostomy. Data regarding CDI incidence, preoperative status, perioperative management, and clinical outcomes were collected. CDI positivity was determined by direct real-time PCR and enzyme-linked fluorescent assays for detecting toxin A and B.Statistical analyses were computed for CDI risk factors.

**Results:**

A total of 1270 patients were included and 208 patients were tested for CDI owing to colitis-related symptoms. The incidence of CDI was 3.6 per cent (46 patients). Multivariable analysis for CDI risk factors identified adjuvant chemotherapy (hazard ratio (HR) 2.28; *P* = 0.034) and colorectal anastomosis leakage prior to CDI (HR 3.75; *P* = 0.008). Finally, patients with CDI showed higher colorectal anastomosis leakage risk in multivariable analysis after ileostomy closure (HR 6.922; *P* = 0.001).

**Conclusion:**

Patients with CDI presented with a significantly higher rate of colorectal anastomosis leakage prior to ileostomy closure.

## Teaser

Adjuvant chemotherapy and colorectal anastomosis leakage increase risk of *Clostridium difficile* infection. Patients with *Clostridium difficile* infection also have a higher risk of anastomosis leakage after rectal surgery.

## Introduction

*Clostridium difficile* infection (CDI) is the most common healthcare-related colitis and results in longer hospitalizations and therefore increased costs^1^. Originally named *Bacillus difficilis* by Hall and O’Toole, owing to the difficulty they faced in isolating and culturing the bacteria^2,3^, *C. difficile* contains two large exotoxins (TcdA and TcdB) that induce the symptoms related to colitis such as diarrhoea, fever, and abdominal pain^4^.

From the 1990s to 2006, the incidence of CDI increased in the USA by threefold (84 cases per 100 000 population in 2005), and deaths caused by CDI in England increased eightfold^3,5,6^. The frequency of this endemic infection, including sporadic outbreaks, seems to have stabilized in the past decade; a decreased CDI burden was evident in the USA from 2011 to 2017 due to a decline in healthcare-associated infections^7^. Notably, however, the CDI rate is still consistently reported to be much higher after colorectal surgery *versus* the healthy population (1 to 2.2 per cent), and is twofold more likely following colorectal procedures than after surgeries not involving the gastrointestinal tract^8^. Furthermore, patients who undergo ileostomy closure have a reported CDI incidence of up to 4 per cent^9–11^.

Other than the longer duration hospital stay and higher financial costs^12^, postoperative morbidities related to CDI have been described in several studies, and a possible but as yet unconfirmed association with postoperative anastomosis leakage has been suggested. Recent studies have described a leak rate of 7 to 45 per cent in CDI-positive patients *versus* 2 to 4 per cent in negative patients who have received colorectal surgery^13–16^. This issue has generated more interest among clinicians, but additional clinical data are now needed to validate this association properly.

The primary aim of this study was to determine the incidence of CDI that develops following ileostomy closure in patients who underwent rectal cancer surgery; secondly, the study aimed to assess the rate of post-ileostomy closure CDI in patients who presented leakage at the original colorectal anastomosis site.

## Methods

### Patient selection

Consecutive patients with rectal cancer who underwent ileostomy closure between January 2015 and December 2019 at Asan Medical Center (a tertiary referral hospital affiliated to Ulsan University of Medicine, which performs more than 3500 colorectal surgeries annually), Seoul, Republic of Korea, were reviewed. These patients had received a diverting ileostomy either prior to or simultaneously with the surgical resection of rectal cancer, or during the postoperative course after primary surgery due to complications such as anastomosis failure. Patients who received palliative surgery were excluded from the cohort, as were any cases who underwent a total colectomy or total proctocolectomy as the presence of remnant colon can be a distinct influence on the microbiome. The hospital medical records were reviewed retrospectively to collect the relevant patient data for the analyses, including age, sex, BMI, alcohol or smoking history, diabetic mellitus (DM), hypertension (HTN), laboratory results (including haemoglobulin, albumin, and creatinine), perioperative neoadjuvant and adjuvant treatment, administered prophylactic antibiotics, and postoperative complications.

The study is reported in accordance with the STROBE guidelines and was approved by the Institutional Review Board of Asan Medical Center (approval number: 2020-0279)^17^. The requirement for patient informed consent was waived by the review board.

### Patient treatments

All patients received curative surgery for rectal cancer. The types of surgery used in this population included low anterior resection (LAR) and ultra-low anterior resection (uLAR). Patients are usually treated using minimal invasive surgery, either laparoscopic or robotic. Patients unfit for minimal invasive surgery for bulky tumours were operated with low midline incisions. The standard method of anastomosis was done by double stapling in which distal rectum is transected by a linear stapler and anastomosed to proximal colon with a circular stapler. When necessary, colo-anal anastomosis was performed manually.

**Fig. 1:**
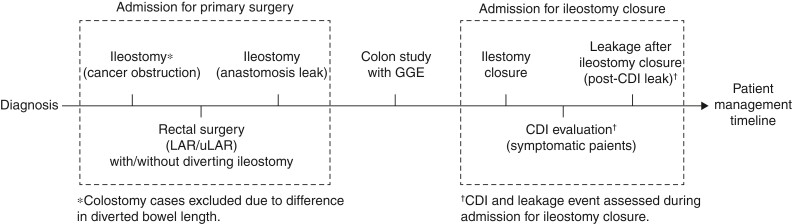
Schematic timeline of patient management

GGE, gastrograffin enema; CDI, *Clostridium difficile* infection; LAR, lower anterior resection; uLAR, ultra-low anterior resection.

Patients who did not receive adjuvant chemotherapy were scheduled for ileostomy closure at 3 months after the primary surgery. Candidates for adjuvant chemotherapy were scheduled for ileostomy closure after the end of the regimen, which was generally 6 to 7 months after the primary surgery. All patients received contrast enema with gastrografin to assess the primary colorectal anastomosis. If there were signs of leakage in the contrast enema, ileostomy closure was postponed until future colon enema study showed no evidence of leakage. Patients were admitted 1 day prior to undergoing ileostomy closure and were subjected to midnight fasting only. On the day of the closure procedure, cefotetan or cefoxitin prophylactic antibiotics were given 30 minutes before the skin incision. The routine postoperative course following ileostomy closure included sips of water on postoperative day 1, a liquid diet on day 2, and a soft diet on days 3 to 4.Patients were considered for discharge when the soft diet was tolerable, with no sign of complications. For patients diagnosed with anastomosis leakage, a combination of third-generation cephalosporin with metronidazole, piperacillin/tazobactam, or vancomycin with meropenem were selected for empirical antibiotic treatment. A schematic timeline of patient management is depicted in *Fig. 1*.

### Assessment and management of *Clostridium difficile* infection

Patients were tested for a possible CDI when a daily excessive watery diarrhoea was observed in the postoperative admission period after ileostomy closure. In our centre, diarrhoea less than 10 times a day is considered natural after ileostomy closure. Patients with less prominent diarrhoea were also tested for CDI if they experienced fever, abdominal distention, ileus, or any other combination of symptoms that are not typical of low anterior resection syndrome. The two diagnostic modalities used for CDI were direct real-time PCR and enzyme-linked fluorescent assay for detecting toxin A and B in anaerobic cultures.

Oral metronidazole (500 mg three times daily) was the treatment of choice for patients diagnosed with CDI. Although oral vancomycin is the first-choice treatment recommended by the guidelines of Infectious Diseases Society of America^18^, oral metronidazole is recommended first choice in South Korea for cost-related reasons. However, oral vancomycin is used in refractory CDI patients who show a poor response to metronidazole.

### Statistical analysis

Categorical variables were analysed with a χ^2^ test. Continuous variables were expressed as the mean (s.d.) and analysed using ANOVA with a Tukey *post hoc* multiple comparison test to determine statistically significant differences between untested patients, patients who tested negative for CDI, and patients who tested positive for CDI. Multivariable analysis with a binary logistic regression model was used to identify independent risk factors for CDI and for leakage at colorectal anastomosis after ileostomy closure. The variables included were factors considered to have clinical relevance to CDI or leakage, and those with a *P* value < 0.1 in the univariable analysis. An abnormal BMI was defined as less than 18.5 or 25 or more, in accordance with the guidelines of the Korean Society for the Study of Obesity^19^, and abnormal serum results were defined in accordance with Asan Medical Center criteria as follows: anaemia (serum haemoglobin < 12 g/dL), hypoalbuminemia (serum albumin < 3.5 g/dL), and high creatinine (serum creatinine ≥ 1.4 mg/dL). All statistical analyses were performed using SPSS^®^ version 21.0 (IBM, Armonk, NY, USA), with *P* values < 0.05 considered to indicate statistical significance.

## Results

### Clinical characteristics of study patients

Of the 1270 patients included in the study total cohort, 208 (16.4 per cent) were tested for CDI and 46 (3.6 per cent) showed a positive result. Almost all of the treated cancers in the cohort were adenocarcinomas (1254 patients), but a small number of cases with other malignancies such as neuroendocrine tumours (six patients) and gastrointestinal stromal tumours (10 patients) were also included. The mean age of the total patient population was 59.81 (±11.3) and 870 (68.5 per cent) patients were male. A LAR was performed in 326 (25.7 per cent) patients, and a uLAR in the remaining 944 (74.3 per cent) patients. Among the study population, leakage at colorectal anastomosis occurred in 71 (5.6 per cent) patients. In 50 (3.9 per cent) of these cases, this leakage was detected during the postoperative course just after the primary resection and anastomosis, and a diverting ileostomy was performed accordingly. The mean interval from the ileostomy diversion to ileostomy closure was 6.5 (±2.7) months. The mean duration of hospital stay for ileostomy closure was 6.73 (±4.2) days. The prophylactic antibiotics used included cefoxitin in 965 (76.0 per cent), cefotetan in 289 (22.8 per cent), ciprofloxacin in eight (0.6 per cent), and ceftriaxone in eight (0.6 per cent) patients. In 25 (2.0 per cent) cases, colorectal anastomosis leakage occurred after the ileostomy closure.

### Risk factors for *Clostridium difficile* infection

Age, BMI, alcohol or smoking histories, DM, HTN, and serum haemoglobulin, albumin, and creatinine levels were comparable between the untested, the CDI-negative, and CDI-positive groups. Preoperative chemoradiotherapy was given to 770 (62.9 per cent) cases in the CDI-negative group (untested + tested negative cases) *versus* 23 patients (50 per cent) in the positive group. Adjuvant chemotherapy was given to 36 (78.3 per cent) patients in the CDI-positive group *versus* 852 cases (69.6 per cent) in the negative group (untested + tested negative cases), but this was not statistically significant (*P* = 0.417). Untested patients were more frequently administered with prophylactic cefotetan than their tested counterparts, but the use of different prophylactic antibiotics was comparable between the CDI-negative and CDI-positive groups. The interval between the generation and closure of the ileostomy did not differ between any of the groups (*P* = 0.231) (*Table 1*).

**Table 1:** Clinical characteristics of the study patients

	Not tested	CDI (−)	CDI (+)	*P*
	(*n* = 1062)	(*n* = 162)	(*n* = 46)	
**Sex**				0.004
Male	709 (66.8)	129 (79.6)	32 (69.6)	
Female	353 (33.2)	33 (20.4)	14 (30.4)	
**Mean (s.d.) age (years)**	60.0 (11.3)	58.56 (10.8)	59.85 (12.1)	0.296
**Mean (s.d.) BMI (kg/m^2^)**	23.93 (3.2)	23.79 (3.4)	23.39 (3.4)	0.491
**Alcohol consumption**	560 (52.8)	95 (58.6)	21 (45.7)	0.217
**Smoking history**	532 (50.1)	89 (54.)	20 (43.5)	0.327
**Diabetes mellitus**	172 (16.2)	30 (18.5)	4 (8.7)	0.280
**Hypertension**	374 (35.2)	57 (35.2)	14 (30.4)	0.801
**Mean (s.d.) haemoglobin**	12.68 (1.6)	12.9 (1.5)	12.59 (1.7)	0.244
**Mean (s.d.) albumin**	3.78 (0.3)	3.81 (0.4)	3.69 (0.4)	0.131
**Mean (s.d.) creatinine**	0.89 (0.4)	0.92 (0.3)	0.98 (0.8)	0.185
**PCRT**	666 (62.7)	104 (64.2)	23 (50.0)	0.194
**Adjuvant chemotherapy**	737 (69.4)	115 (71.0)	36 (78.3)	0.417
**Type of surgery**				0.130
LAR	261 (24.6)	51 (31.5)	14 (30.4)	
uLAR	801 (75.4)	111 (68.5)	32 (69.6)	
**Prophylactic antibiotics**				< 0.001
Cefotetan	276 (26.0)	11 (6.8)	2 (4.3)	
Cefoxitin	771 (72.6)	150 (92.6)	44 (95.7)	
Ceftriaxone	7 (0.7)	1 (0.6)	0 (0)	
Ciprofloxacin	8 (0.8)	0 (0)	0 (0)	
**Mean (s.d.) time to closure (months)**	6.45 (2.6)	6.81 (2.6)	6.72 (3.1)	0.231
**Mean (s.d.) duration of hospital stay (days)**	6.03 (2.5)	10.19 (7.9)	10.78 (7.4)	< 0.001
Leak*	45 (4.2)	16 (9.9)	10 (21.7)	< 0.001
Pre-CDI leak	33 (3.1)	11 (6.8)	6 (13.0)	< 0.001
Post-CDI leak	14 (1.3)	7 (4.3)	4 (8.7)	< 0.001

Values are presented as a n (%) or as a mean (s.d.). *Leakage was assessed for anastomosis between colon and rectum. CDI, *Clostridium difficile* infection; PCRT, preoperative chemoradiotherapy; LAR, low anterior resection; uLAR, ultra-low anterior resection.

Confounding factors were adjusted in subsequent multivariable analysis for prognostic indicators of CDI. Patients who experienced anastomosis leakage prior to the detection of CDI presented with a significantly higher risk (hazard risk (HR) 3.753; *P* = 0.008) of developing this infection. Adjuvant chemotherapy was also found to be associated with a higher risk of CDI (HR 2.276; *P* = 0.034) (*Table 2*).

**Table 2:** Multivariable analyses of prognostic factors for *Clostridium difficile* infection

Variable	Hazard ratio	95% confidence interval	*P*
**Age ≥ 65 years**	1.673	0.863–3.244	0.128
**Sex, male**	0.818	0.3663–1.829	0.625
**Abnormal BMI (<18.5 or ≥ 25 kg/m^2^)**	0.843	0.4473–1.591	0.599
**Alcohol consumption**	0.850	0.4033–1.795	0.670
**Smoking history**	0.680	0.3073–1.507	0.342
**Diabetic mellitus**	0.511	0.1743–1.500	0.222
**Hypertension**	0.778	0.3853–1.571	0.484
**Serum haemoglobin < 12**	0.558	0.2653–1.176	0.125
**Serum albumin < 3.5**	1.894	0.8923–4.020	0.096
**Serum creatinine ≥1.4**	0.996	0.2133–4.654	0.996
**PCRT**	0.552	0.2943–1.037	0.065
**Adjuvant chemotherapy**	2.276	1.0643–4.870	0.034
**Pre-CDI leak** *	3.753	1.4103–9.990	0.008

* Leakage was assessed for anastomosis between colon and rectum. PCRT, preoperative chemoradiotherapy; CDI, *Clostridium difficile* infection.

### Clinical outcomes of a *Clostridium difficile* infection

The mean duration of hospitalization was significantly longer for the patients who tested positive for CDI compared with the other groups in the study cohort (10.78 ± 7.4 *versus* 6.58 ± 3.9 days; *P* < 0.001). Notably, however, the duration of hospitalization was similar between the CDI-positive and CDI-negative patients among the tested population (10.78 ± 7.4 *versus* 10.19 ± 7.9; *P* = 0.626). CDI positivity was found to be associated with a higher incidence of colorectal anastomosis leakage *versus* either the untested or tested negative groups (*P* < 0.001 and *P* = 0.032, respectively) (*Table 1*). Patients who experienced anastomosis leakage prior to an ileostomy closure were treated with a wide range of empirical antibiotics. Among the 50 cases in the series in this category, 16 patients were treated with piperacillin/tazobactam, 15 with imipenem, 11 with third-generation cephalosporin with or without metronidazole, and eight with combinations of antibiotics, including vancomycin, tigecycline, meropenem, clindamycin, and moxifloxacin. Five patients (10.9 per cent) did not respond to oral metronidazole and were switched to oral vancomycin, subsequently showing improvement in their symptoms.

**Fig. 2:**
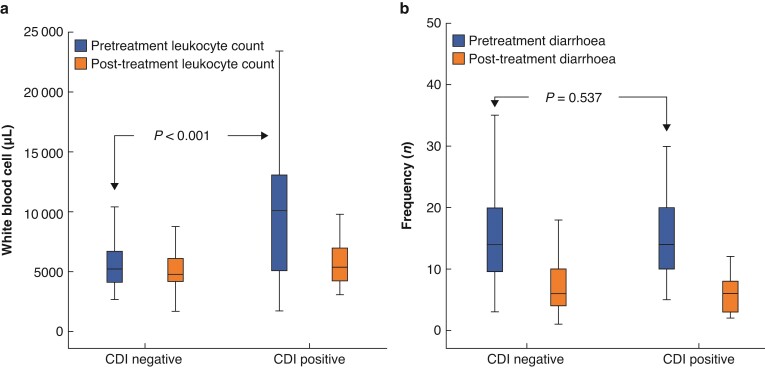
**a** Box plot showing significantly increased leukocyte counts in *Clostridium difficile* infection (CDI)-positive patients prior to undergoing treatment, which were improved by oral metronidazole. CDI-negative patients did not present with leukocyte count increase. **b** The daily number of diarrhoea events was comparable between the CDI-negative and CDI-positive patients both pre- and post-treatment with oral metronidazole.

Among the 208 patients tested for CDI after ileostomy closure, an increased white blood cell count at the time of symptom onset was evident in 23 cases (50 per cent) with a mean value of 9921/µl in CDI-positive patients. Patients who tested negative for CDI presented with leukocytosis in 12 cases (7.4 per cent) with a mean value of 5810/µl. CDI-positive patients presented with a fever above 38.0°C in 23 (50 per cent) cases, while fever was noted in only 28 (17.3 per cent) patients who tested negative for CDI. After treatment with oral metronidazole, CDI-positive patients showed improvement in relation to their leukocytosis with a mean value of 5730/µl, while CDI-negative patients showed a value of 5127/µl (*Fig. 2a*). The mean daily frequency of diarrhoea prior to receiving oral metronidazole did not differ between the CDI-positive and CDI-negative groups (15.1 ± 8.7 and 14.3 ± 7.2 times, respectively). Both groups showed a decreased incidence of diarrhoea after taking oral metronidazole (6.3 ± 3.4 and 7.6 ± 5.4, respectively; *Fig. 2b*). Prominent symptoms of ileus (abdominal distention, nausea/vomiting) were identified in 21 CDI-positive patients (45.7 per cent) and were accompanied by a distinctive pattern of bowel dilatation, displaying a continued loss of haustra from the descending colon to the anastomosis site. CDI-negative patients presented with signs of ileus in 50 cases (30.9 per cent; *P* = 0.117).

Potential risk factors for anastomosis leakage after ileostomy closure were analysed in the multivariable analyses (*Table 3*). Patients who tested positive for CDI show significantly higher risk of anastomosis leakage after ileostomy closure (HR 6.922; *P* = 0.001).

**Table 3:** Multivariable analyses of risk factors for leakage after ileostomy closure*

Variable	Hazard ratio	95% confidence interval	*P*
**Age ≥65 (years)**	0.271	0.091–0.809	0.019
**Sex, male**	0.382	0.122–1.195	0.098
**Abnormal BMI (< 18.5 or ≥ 25 kg/m^2^)**	0.551	0.220–1.377	0.202
**Diabetic mellitus**	2.103	0.799–5.539	0.132
**Hypertension**	1.951	0.804–4.734	0.139
**Serum haemoglobin < 12**	1.132	0.415–3.086	0.809
**Serum albumin < 3.5**	1.921	0.654–5.645	0.235
**Serum creatinine ≥ 1.4**	0.390	0.045–3.384	0.393
**PCRT**	1.379	0.529–3.596	0.511
**Adjuvant chemotherapy**	0.608	0.233–1.583	0.308
**uLAR (*versus* LAR)**	1.547	0.538–4.454	0.418
**CDI-positive**	6.922	2.115–22.654	0.001

PCRT, preoperative chemoradiotherapy; uLAR, ultra-low anterior resection; LAR, low anterior resection; CDI, *Clostridium difficile* infection. *Leakage was assessed for anastomosis between colon and rectum.

## Discussion

The present study findings indicate that anastomosis leakage prior to ileostomy closure is associated with a higher CDI rate after ileostomy closure, and that patients who develop CDI are more vulnerable to leakage at primary anastomosis after their ileostomy closure. While previous studies related to risk factors for CDI have reported comorbidities as significant variables, including DM, heart conditions, chronic renal disease, and old age, our current results revealed that only adjuvant chemotherapy and anastomosis leakage are independent risk factors for CDI.

Among the 46 cases in the current study cohort diagnosed with CDI, six (13 per cent) and four patients (8.7 per cent) experienced anastomosis leakage before and after ileostomy closure, respectively. All patients in the current series who received a diverting ileostomy due to anastomosis failure received extensive empirical antibiotic treatment. This could explain the high incidence of CDI in these cases as a previous exposure to antibiotics is the most well-known risk factor for this infeciton^20,21^. A previous study reported a 4.2 per cent rate of CDI after ileostomy closure *versus* 2.1 per cent for a right hemicolectomy and 1 per cent for an anterior resection. One of the reasons for the higher rate of CDI after ileostomy closure was anticipated to be the previous surgical procedure that the patients received before ileostomy closure that required antibiotic use^9^. Prior studies have also indicated that changes can occur in the bowel microbiome after stool diversion, which need to be considered^22^.

With regard to patients who experienced anastomosis leakage after ileostomy closure, it is more difficult to determine in these cases whether CDI was the cause of the leakage. Colorectal anastomosis leak in patients with diverting ileostomy could be inapparent until ileostomy closure is performed when only then faecal material may cause clinical symptoms. Therefore, one should be cautious in suggesting CDI as the cause of leakage. However, there are increasing reports from various studies supporting this issue. One study reported a 6.69 per cent anastomosis leakage rate in patients with postoperative CDI *versus* 3.06 per cent in CDI-negative cases from a total study population of 56 631 patients who had undergone a colectomy^14^. Another study described 19 cases of anastomosis leakage out of 320 patients, of which 13 cases were CDI-positive (*P* < 0.001)^15^. A Japanese group also reported seven patients (3.8 per cent) with anastomosis leakage from a cohort of 185 colorectal surgery cases and indicated that CDI was significantly associated with leakage (*P* = 0.001). Finally, few case reports of anastomosis rupture related to CDI have been published^13,16^. Although the number of cases of anastomosis leakage after ileostomy closure is very small, the current findings indicate a higher rate of leakage in CDI-positive patients *versus* negative cases (8.7 per cent *versus* 1.4 per cent, respectively). Symptomatic *C. difficile* colonization is known to induce inflammation, secretions, bowel dilatation, and oedemas. The activity of *C. difficile* toxins has also been shown to stimulate an inflammatory host response that induces the degradation of collagen and other components of the extracellular matrix^23,24^. For these reasons, concerns regarding integrity of the anastomosis are well-founded in patients diagnosed with CDI, and patients at risk of these infections must be closely monitored.

There have been extensive studies to date on the risk factors for CDI such as old age, DM, smoking history, use of steroids, impaired kidney function, and a poor nutrition status^25–27^. Moreover, in an era where chemotherapy has become a mainstay treatment for patients with cancer, adjuvant chemotherapy is reported to increase the risk of CDI in oncology patients^28,29^. These results are consistent with the present study’s findings, which indicate adjuvant chemotherapy as one of two independent risk factors for the development of CDI. Although no clear risk factors for CDI other than adjuvant chemotherapy can yet be proposed for selecting candidates for a more thorough observation, conventional risk factors suggested by previous studies should also be considered when patients experience excessive diarrhoea, leukocytosis, fever, or any other symptoms related to colitis.

This study had some limitations, principally stemming from its retrospective nature. Although the medical records of the study subjects were reviewed as completely as possible, data for some patients were missing. Also, the heterogenous nature of management for involved patients in testing for CDI, either the indications used for performing tests or the testing modalities used, is likely to have influenced the incidence rate of CDI in the present cohort. Nevertheless, the current data indicate a clear trend regarding the onset of CDI in a single tertiary centre among a homogenous group of patients with rectal cancer who had all undergone an ileostomy closure.

*Disclosure*. The authors declare no conflict of interest.

## Data availability

Raw data were generated at Asan Medical Center. Derived data supporting the findings of this study are available on request from the corresponding author.
